# Microbe Associated Molecular Pattern Signaling in Guard Cells

**DOI:** 10.3389/fpls.2016.00583

**Published:** 2016-05-04

**Authors:** Wenxiu Ye, Yoshiyuki Murata

**Affiliations:** Graduate School of Environmental and Life Science, Okayama UniversityOkayama, Japan

**Keywords:** guard cell, microbe-associated molecular patterns, reactive oxygen species, ion channels, Ca^2+^ signaling, mitogen-activated protein kinase, open stomata 1, Ca^2+^-dependent protein kinase

## Abstract

Stomata, formed by pairs of guard cells in the epidermis of terrestrial plants, regulate gas exchange, thus playing a critical role in plant growth and stress responses. As natural openings, stomata are exploited by microbes as an entry route. Recent studies reveal that plants close stomata upon guard cell perception of molecular signatures from microbes, microbe associated molecular patterns (MAMPs), to prevent microbe invasion. The perception of MAMPs induces signal transduction including recruitment of second messengers, such as Ca^2+^ and H_2_O_2_, phosphorylation events, and change of transporter activity, leading to stomatal movement. In the present review, we summarize recent findings in signaling underlying MAMP-induced stomatal movement by comparing with other signalings.

## Introduction

Stomata are microscopic pores formed by pairs of guard cells in the shoot epidermis of plants and regulate gas exchange, notably CO_2_ uptake for photosynthesis and water loss during transpiration, thus playing a critical role in plant growth and stress responses such as drought stress. As natural openings, stomata are exploited as a major entry route by a wide range of microbes including bacteria, oomycetes, and fungi (Grimmer et al., 2012; Sawinski et al., 2013). On the other hand, plants proactively induce stomatal closure and inhibit stomatal opening to prevent microbe invasion, which is later termed as stomatal immunity (Melotto et al., 2008; Sawinski et al., 2013). Stomatal immunity is closely related to susceptibility to a variety of pathogenic microbe infections. For example, defection in stomatal closure in response to pathogenic bacteria increased infection in *Arabidopsis* (Melotto et al., 2006; Singh et al., 2012), while pre-closing stomata by abscisic acid (ABA) reduced infection in grapevine by oomycete, *Plasmopara viticola* (Allègre et al., 2009).

Microbe-associated molecular patterns (MAMPs) are molecular signatures that are highly conserved in whole classes of microbes but are absent from the host, such as chitin for fungi and flagellin for bacteria (Boller and Felix, 2009). Recognition of each MAMP is performed by specific surface-localized receptors containing various ligand-binding ectodomains in plants, which are termed as pattern-recognition receptors (PRRs). The perception of MAMPs by PRRs triggers signaling converging to common responses, such as ion fluxes including Ca^2+^ influx, K^+^ eﬄux, and anion eﬄux, production of reactive oxygen species (ROS) and phosphorylation events, which are critical for plant innate immunity (Boller and Felix, 2009; Zipfel, 2014). Recent studies revealed that several MAMPs induce stomatal closure and inhibit stomatal opening, including flg22 (a conserved 22-amino-acid peptide near the N terminus of bacterial flagellin (Melotto et al., 2006; Zhang et al., 2008). Further results showed that plants with loss-of-function of the PRR for flg22, FLAGELLIN-SENSITIVE 2 (FLS2), did not close stomata in response to flg22 and coronatine-deficient *Pseudomonas syringae* pv *tomato* (*Pst*) strain DC3000, *Pst* DC3118 (Melotto et al., 2006; Zeng and He, 2010). As a result, the mutant is more susceptible to *Pst* DC3118 than wild type. These results indicate that plants mainly sense MAMPs to induce stomatal closure to prevent microbe invasion. The past decade has seen increasing efforts in elucidating MAMP signaling in guard cells and exciting findings every year. Several excellent reviews have been published covering this topic (Melotto et al., 2008; Zeng et al., 2010; Sawinski et al., 2013; McLachlan et al., 2014; Arnaud and Hwang, 2015). In the present review, we concentrate on the current knowledge of MAMP signaling in guard cells and discuss the latest findings by comparing with other signalings.

## Abscisic Acid Signaling in Guard Cells

Phytohormones play critical roles in regulating stomatal movement. Almost all the phytohormones are reported to be involved in stomatal movement, among which ABA, methyl jasmonic (MeJA), and salicylic acid (SA) are believed to induce stomatal closure in various plants (Acharya and Assmann, 2009; Murata et al., 2015). Particularly, mechanism of stomatal movement has been well characterized in the context of ABA signaling in guard cells. In this section, we briefly overview ABA signaling in *Arabidopsis* guard cells. For details on this topic, we refer readers to excellent reviews (Hubbard et al., 2010; Kim et al., 2010; Joshi-Saha et al., 2011).

Abscisic acid is mainly produced in response to drought stress and the ABA synthesized in guard cells plays a critical role in regulation of stomatal movement (Bauer et al., 2013). An Snf1-related protein kinase 2 (SnRK2), SnRK2.6 also known as Open stomata 1 (OST1), is a Ca^2+^-independent protein kinase and an essential positive regulator in ABA signaling in *Arabidopsis* guard cells. In resting condition, OST1 kinase activity is inhibited by clade A Type 2C protein phosphatases (PP2Cs). Upon ABA perception, the interaction of ABA receptors, PYR/PYL/RCAR, and PP2Cs releases the inhibition of OST1, resulting in increment of OST1 kinase activity (Cutler et al., 2010; Hubbard et al., 2010; Joshi-Saha et al., 2011). In guard cell ABA signaling, OST1 is essential for recruitment of second messengers, such as H_2_O_2_, NO, and Ca^2+^, which are important for regulation of transporters in the plasma membrane including S-type anion channels and H^+^-ATPases (Mustilli et al., 2002; Bright et al., 2006; Acharya et al., 2013; Yin et al., 2013). OST1 has been reported to directly regulate ion channels including S-type anion channel SLAC1 (Geiger et al., 2009; Lee et al., 2009; Brandt et al., 2012), R-type anion channel ALMT12 (Meyer et al., 2010; Sasaki et al., 2010; Imes et al., 2013), inward-rectifying K^+^ channels (*K*_in_ channels) KAT1 (Sato et al., 2009; Siegel et al., 2009; Uraji et al., 2012), K^+^ uptake transporter 6 (KUP6; Osakabe et al., 2013), vacuolar anion exchanger CLCa (Wege et al., 2014) and Plasma membrane Intrinsic Protein 2;1 (PIP2;1; Grondin et al., 2015). Downstream components are also involved in ABA regulation of transporters including several Ca^2+^-dependent protein kinases (CDPK), CPK3, CPK4, CPK5, CPK6, CPK10, CPK11, and CPK23 (Mori et al., 2006; Zhu et al., 2007; Zou et al., 2010; Brandt et al., 2015), and mitogen-activated kinases (MAPKs), MPK9 and MPK12 (Jammes et al., 2009). The regulation of transporters by ABA, especially the activation of anion channels and suppression of H^+^-ATPases, causes depolarization of plasma membrane. For open stomata, this leads to eﬄux of anion, K^+^ and water, resulting in stomatal closure. For closed stomata, this keeps stomata closed in response to light.

## MAMPs Known to Induce Stomatal Closure

A wide range of MAMPs from bacteria, fungi and oomycetes have been identified and characterized (Zipfel, 2014). New types of MAMPs are emerging such as peptides from Necrosis and ethylene-inducing peptide 1 (Nep1)-like proteins (Oome et al., 2014) and glycoside hydrolase family 12 (GH12) protein (Ma et al., 2015). The well-known MAMPs that induce stomatal movement are bacterium-derived MAMPs including flg22, elf18/elf26 (the first 18/26-amino-acid peptide of the N terminus of bacterial elongation factor Tu) and bacterial lipopolysaccharide (LPS), and fungus-derived MAMPs including chitin, chitosan and YEL. β-1→3-linked glucans are major components in oomycete cell wall and have been used to study stomatal movement (Allègre et al., 2009; Fu et al., 2011; Robinson and Bostock, 2014). In this section, we briefly introduce the molecular nature and perception of these MAMPs. For detail, we refer readers to excellent reviews (Boller and Felix, 2009; Zipfel, 2014; Sánchez-Vallet et al., 2015; Shinya et al., 2015).

### MAMPs Derived from Bacteria

The widely used flg22, QRLSTGSRINSAKDDAAGLQIA, is an epitope in the flagellin of *P. syringae* pv. *tabaci* 6605, and induces stomatal closure and inhibits light-trigged stomatal opening in *Arabidopsis* (Melotto et al., 2006; Zhang et al., 2008). Though this epitope is widely conserved, studies have shown that variations of this epitope that are not sensed by *Arabidopsis* exist in several pathogenic bacteria (Pfund et al., 2004; Robatzek et al., 2006). The flg22 used here only refers to the one from *P. syringae* pv. *tabaci* 6605, which has been used in most of studies on stomatal movement. The perception of flg22 is mediated by a plasma membrane-localized leucine-rich repeat type receptor-like kinase (LRR-RLK), Flagellin-sensitive 2 (FLS2; Gomez-Gomez and Boller, 2000), which is expressed in guard cells (Melotto et al., 2006; Beck et al., 2014). Loss-of-function mutation of *FLS2* abolishes flg22-induced stomatal movement (Melotto et al., 2006; Zhang et al., 2008). Recent studies identified another epitope in the flagellin from various *P. syringae* pv. *tomato*, 28 amino acid long peptides (flgII-28), which induces stomatal closure in tomato (Cai et al., 2011). However, the PRR for flgII-28 remains to be identified.

Elongation factor is one of the most abundant proteins in bacteria. The widely used elf18, *N*-acetylated SKEKFERTKPHVNVGTIG, and elf26, *N*-acetylated SKEKFERTKPHVNVGTIGHVDHGKTT, are the bioactive N terminals of elongation factor with the first 18 and 26 amino acid residues from *Escherichia coli* and both induce stomatal closure in *Arabidopsis* (Zeng and He, 2010). The PRR for these two peptides in *Arabidopsis* is Elongation factor Tu receptor (EFR), which is also an LRR-RLK expressed in guard cells (Zipfel et al., 2006; Liu et al., 2009). Plants outside of the Brassicaceae are believed not to respond to this epitope of EF-Tu due to lack of EFR (Zipfel et al., 2006). Recently, a new epitope from the middle region comprising Lys176 to Gly225 of the *Acidovorax avenae* EF-Tu, termed as EFa50, is identified as a MAMP sensed by rice (Furukawa et al., 2014). It remains to be clarified whether EFa50 regulates stomatal movement in rice.

Upon binding of flg22 or elf18/elf26, FLS2 or EFR forms receptor complexes with LRR-RLKs belonging to Somatic-embryogenesis receptor-like kinase (SERK) family, among which Brassinosteroid insensitive1-associated kinase1/SERK3 (BAK1/SERK3) plays a dominant role (Chinchilla et al., 2007; Roux et al., 2011). Structure data revealed that the C-terminal region of flg22 functions as a molecular glue between ectodomains of FLS2 and BAK1 (Sun et al., 2013). Recent studies revealed that receptor-like cytoplasmic kinases (RLCKs) are direct substrates of the receptors complexes and transduce the signal of MAMPs to downstream events (Macho and Zipfel, 2014). A RLCKs, Botrytisinduced kinase1 (BIK1), is essential for stomatal closure induced by flg22 but not ABA (Li et al., 2014).

Lipopolysaccharides are characteristic components of Gram-negative bacteria and composed of conserved lipid A, core oligosaccharide regions and a long-chain poly saccharide (the O antigen) that can have variable composition, length, and branching of its carbohydrate subunits. Due to variety in structure and easy contamination during preparation, LPS used in many of the plant researches can be consider a mixture of MAMPs and is derived from human pathogen *P. aeruginosa.* The LPS induces stomatal closure in *Arabidopsis* and tomato (Melotto et al., 2006; Liu et al., 2009; Desclos-Theveniau et al., 2012). Lipopolysaccharides from *E. coli* O55:B5 induced stomatal closure in *Arabidopsis* (Melotto et al., 2006). The PRRs for LPS are still under investigation. It has been shown that plants sense the O antigen, core oligosaccharide and lipid A (Bedini et al., 2005; Silipo et al., 2005). Recent studies further revealed that a bulb-type (B-type) lectin S-domain (SD)-1 RLK, Lipooligosaccharide-specific reduced elicitation (LORE), functions as a putative PRR for LPS from *Pseudomonas* and *Xanthomonas* species including *P. aeruginosa* by sensing lipid A moiety, and is restricted to the Brassicaceae family of plants (Ranf et al., 2015). Interestingly, *Arabidopsis* responds to LPS from *E. coli* K12 and *E. coli* O111:B4 in an LORE-independent manner (Ranf et al., 2015). These results suggest that multiple PRRs including LORE are involved in LPS-induced stomatal closure.

### MAMPs Derived from Fungi and Oomycetes

Chitin is an insoluble polymer of β-1,4-linked *N*-acetylglucosamineone and one of the major components of fungal cell wall. Chitosan is the deacetylated form of chitin. Caution has to be paid that commercially available chitosan can be only partially deacetylated. For example, some chitosan from Sigma is 75 to 85% deacetylated. Chitin induces stomatal closure in *Arabidopsis* (Lozano-Duran et al., 2014; Bourdais et al., 2015). Chitosan induces stomatal closure and inhibits light-induced stomatal opening in various plant species, such as *Arabidopsis* (Klüsener et al., 2002), tomato (Lee et al., 1999), pea (Srivastava et al., 2009), rapeseed (Li et al., 2009), tobacco (Fu et al., 2011), and barley (Koers et al., 2011). In these studies, both chitin and chitosan are a mixture of polymers with different degree of polymerization (DP). Actually, the degree of polymerization is critical for the plant responses to chitin and chitosan (Kauss et al., 1989; Yamada et al., 1993; Vander et al., 1998; Liu et al., 2012). A current model shows that perception of chitin in *Arabidopsis* involves three lysin motif type RLKs (LysM-RLKs), Lysin motif receptor kinase 5 (LYK5), LYK4 and chitin elicitor receptor kinase 1 (CERK1) (Miya et al., 2007; Cao et al., 2014). It has been reported that LYK5 has much higher affinity to chitin than CERK1 does, interacts with CERK1, and is indispensable for chitin-triggered CERK1 phosphorylation (Cao et al., 2014). These results suggest that LYK5 and CERK1 form a receptor complex for chitin. Recent studies also identified several RLCKs as downstream components of the receptor complex (Zhang et al., 2010; Shinya et al., 2014). For the perception of chitosan, high DP of chitosan seems to be critical (Kauss et al., 1989; Vander et al., 1998; Iriti and Faoro, 2009). Chitosan oligomers only weakly bind to CERK1 and is unlikely to induce several plant responses including ROS production (Kauss et al., 1989; Vander et al., 1998; Petutschnig et al., 2010). It is further suggested that surface charge of fully deacetylated chitosan polymers is responsible for their effects on plants (Kauss et al., 1989).

Mixtures of β-1→3-linked glucans with different DP have been shown to induce stomatal closure and inhibits stomatal opening in grapevine and tobacco (Allègre et al., 2009; Fu et al., 2011). The strength of stomatal closure induced by the glucans showed dependency on DP, which may reflect the different perceptions of these glucans. However, the molecular mechanism of β-1→3-linked glucan perception is poorly understood.

Elicitors from baker’s yeast (YEL) extracted by ethanol precipitation mainly contains fungal cell wall fraction including mannan, β-1→3-linked glucans, chitin, and glycopeptides, and has been widely used as a fungal MAMP to induce plant immune response including stomatal responses (Hahn and Albersheim, 1978; Schumacher et al., 1987; Gundlach et al., 1992; Blechert et al., 1995; Kollar et al., 1997; Klüsener et al., 2002; Zhao et al., 2004; Ge and Wu, 2005; Khokon et al., 2010a; Salam et al., 2013; Ye et al., 2013b, 2015; Moon et al., 2015; Narusaka et al., 2015). YEL induces stomatal closure and inhibits light-induced stomatal opening in *Arabidopsis* (Klüsener et al., 2002; Khokon et al., 2010a; Salam et al., 2013; Ye et al., 2013b, 2015).

## Core Signaling Events Downstream of Mamp Perception in Guard Cells

Guard cell signaling induced by MAMPs involves in recruitment of second messengers, such as H_2_O_2_, NO, and Ca^2+^, phosphorylation events mediated by CDPKs, OST1, and MAPKs, and changes of transporter activity. In this section, we review the main signaling events upon perception of MAMPs with focus on findings from *Arabidopsis* guard cells.

### Production of Reactive Oxygen Species in Guard Cell MAMP Signaling

Reactive oxygen species, particularly H_2_O_2_, are important second messengers in guard cell signaling induced by abiotic and biotic factors (Pei et al., 2000; Zhang et al., 2001; Khokon et al., 2010a; Hoque et al., 2012; Hossain et al., 2013; Ye et al., 2013a; Kadota et al., 2014; Sobahan et al., 2015). Researches using leaf disks have provided most of the current knowledge of MAMP-induced H_2_O_2_ production. Recent studies further revealed that MAMPs including flg22, elf26, LPS, chitosan, β-1→3-linked glucans and YEL induce accumulation of H_2_O_2_ in guard cells (Desikan et al., 2008; Allègre et al., 2009; Ma W. et al., 2009; Desclos-Theveniau et al., 2012; Ma et al., 2013; Salam et al., 2013; Ye et al., 2015).

**FIGURE 1:**
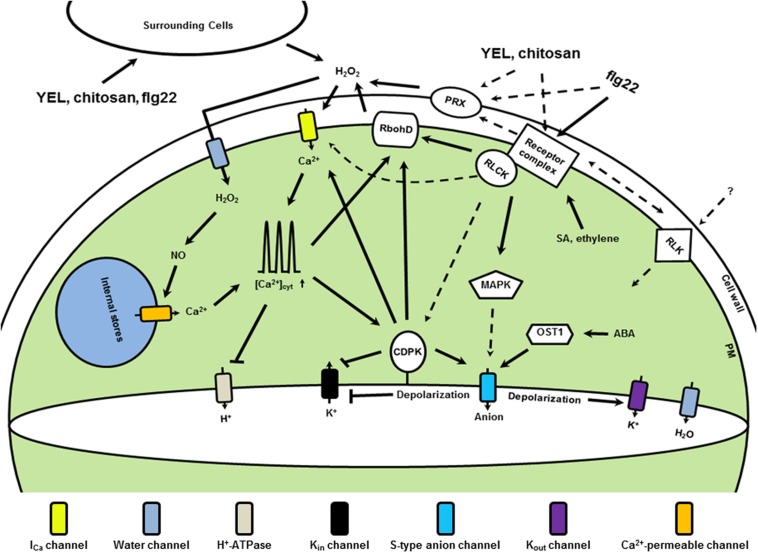
**Simplified microbe associated molecular patterns (MAMP) signaling in *Arabidopsis* guard cells**. Upon perception of MAMPs, such as YEL, chitosan and flg22, receptor complexes are formed in the plasma membrane, which activate receptor-like cytoplasmic kinases (RLCKs). Downstream events are H_2_O_2_ production mediated by RbohD and PRXs, and activation of MAPKs and unidentified I_Ca_ channels. H_2_O_2_ accumulates in the cytosol and induces production of NO, which regulates Ca^2+^ release from internal stores. Elevation of [Ca^2+^]_cyt_ negatively regulates the activity of H^+^-ATPases and activates CDPKs. Regulation by upstream components is also essential for CDPK activation. CDPKs negatively regulate *K*_in_ channel activity but positively regulate *I*_Ca_ channels and S-type anion channels. MAPK and resting OST1 activity are essential for S-type anion channel activation. Ethylene and SA at resting level regulate receptor levels. ABA at resting level may contribute to the resting OST1 activity. Increasing RLKs, such as GHR1, CRKs, and LecRKs, are found to be involved in MAMP signaling, but their activation is largely unknown. Arrowheads designate positive regulation and bars show negative regulation. Dashed lines indicate possible pathways need to be clarified, and question marks unconfirmed components.

One of the main mechanisms involved in H_2_O_2_ production is mediated by plasma membrane-localized NAD(P)H oxidases, RBOHD and RBOHF. NAD(P)H oxidases transfer electrons from cytosolic NAD(P)H to apoplastic oxygen, leading to superoxide production. The superoxide can be converted to H_2_O_2_ through dismutation by unknown superoxide dismutase (Jannat et al., 2011; Suzuki et al., 2011). The produced H_2_O_2_ can accumulate in guard cells through diffusion and water channels (Henzler and Steudle, 2000; Bienert et al., 2007; Grondin et al., 2015). In ABA and MeJA signaling, both RBOHD and RBOHF redundantly regulate H_2_O_2_ production and stomatal closure (Kwak et al., 2003; Suhita et al., 2004; Munemasa et al., 2007). On the other hand, it seems that RBOHD plays a prominent role in flg22- and elf18-induced stomatal closure and H_2_O_2_ production in leaf (Mersmann et al., 2010; Macho et al., 2012; Kadota et al., 2014; **Figure 1**). Regarding the activation of NAD(P)H oxidases, studies have shown that elevation of free cytosolic Ca^2+^ concentration ([Ca^2+^]_cyt_) and phosphorylation by CDPKs are important for flg22- and elf18-induced H_2_O_2_ production in leaf disks (Boudsocq et al., 2010; Dubiella et al., 2013; Kadota et al., 2014), but not for ABA- and MeJA-induced H_2_O_2_ production in guard cells (Suhita et al., 2004; Munemasa et al., 2011; Brandt et al., 2015). Ca^2+^-independent phosphorylation is essential to the activation of NAD(P)H oxidases. In ABA signaling, the Ca^2+^-independent protein kinase, OST1, phosphorylates RBOHF at Ser13 and Ser174 and interacts with RBOHD (Sirichandra et al., 2009; Acharya et al., 2013) and is essential for H_2_O_2_ production in guard cells (Mustilli et al., 2002; Yin et al., 2013). Recent studies revealed that phosphorylation of several phosphorylation sites at the N-terminal part of RBOHD including Ser39 and Ser343 is increased in response to flg22 and elf18, which is independent on the elevation of [Ca^2+^]_cyt_ but dependent on the Ca^2+^-independent activation of RLCKs, particularly BIK1 (Kadota et al., 2014; Li et al., 2014). Further results showed that phosphor-dead mutations of these phosphorylation sites suppress H_2_O_2_ production and stomatal closure induced by flg22 and elf18 and phosphor-mimetic mutations of the sites do not induce H_2_O_2_ production and stomatal closure but complement the flg22- and elf18-induced H_2_O_2_ production and stomatal closure in plants of loss-of-function mutations of *BIK1* and another *RLCK*, *PBL1*. These results indicate that phosphorylation of these sites by RLCKs is essential but not sufficient to induce H_2_O_2_ production in leaf disks. To integrate the Ca^2+^-dependent and -independent regulation of RBOHD, the authors proposed that the BIK1-mediated phosphorylation primes RBOHD activation by increasing the sensitivity to the Ca^2+^-based regulation (Kadota et al., 2014, 2015; Li et al., 2014). Future work is needed to elucidate this hypothesis.

Another important mechanism involved in H_2_O_2_ production in guard cell is mediated by class III peroxidases (PRXs; **Figure 1**). Both internal factors, such as SA (Mori et al., 2001; Khokon et al., 2011), isothiocyanates (ITCs; Hossain et al., 2013; Sobahan et al., 2015) and methylglyoxal (Hoque et al., 2012), and external factors, such as YEL (Khokon et al., 2010a), chitosan (Khokon et al., 2010b), flg22 (O’Brien et al., 2012), and elf26 (O’Brien et al., 2012), induce H_2_O_2_ production mediated by PRXs. Pharmacological and genetic studies further showed that salicylhydroxamic acid (SHAM)-sensitive PRXs but not RBOHD or RBOHF are essential for stomatal closure and H_2_O_2_ production in guard cells induced by SA, ITCs, methylglyoxal, chitosan and YEL (Khokon et al., 2010a,b, 2011; Hoque et al., 2012; Hossain et al., 2013). The chemistry of H_2_O_2_ production by PRXs can be affected by factors including pH and reductants. For example, under an acidic reaction condition, SA itself functions as electron donor to produce superoxide catalyzed by PRXs, which then can be converted to H_2_O_2_ by dismutases (Mori et al., 2001). Under an alkali condition, PRXs can directly utilize electron donors to produce H_2_O_2_, in which it seems that thiol compounds are preferred as the electron donors (Bolwell et al., 1995; Bolwell et al., 2002). While the identification of reductants in a physiological context remains challenging, apoplast alkalization is widely observed in plants treated by MAMPs such as flg22, elf18, and chitin (Felix et al., 1999; Felle et al., 2004; Zipfel et al., 2006; Boller and Felix, 2009). Recent studies have tried to identify the PRXs involved in the H_2_O_2_ production induced by MAMPs. Two PRXs, PRX33, and PRX34, were shown to be involved in H_2_O_2_ production induced by flg22, elf26, and fungal derived MAMPs (Daudi et al., 2012; O’Brien et al., 2012). However, it remains unknown whether these PRXs are involved in stomatal movement.

### Production of Nitric Oxide Induced by MAMPs in Guard Cells

Nitric oxide has been widely shown to be involved in stomatal movement induced by various biotic and abiotic stimuli such as ABA, MeJA, SA, allyl isothiocyanate (AITC), and flg22 (Neill et al., 2002; Melotto et al., 2006; Garcia-Mata and Lamattina, 2007; Munemasa et al., 2007; Khokon et al., 2010a, 2011). Flg22, LPS, YEL and chitosan induce NO production in guard cells, which is required for stomatal closure (Melotto et al., 2006; Srivastava et al., 2009; Khokon et al., 2010a,b). Application of H_2_O_2_ has been shown to induce NO production in *Arabidopsis* guard cells (Bright et al., 2006). It is likely that H_2_O_2_ contributes to NO production induced by MAMPs. Genetic and pharmacological studies have shown that H_2_O_2_ is involved in ABA-induced NO production (Bright et al., 2006). Pharmacological studies have also shown that H_2_O_2_ is involved in NO production induced by YEL and chitosan (Srivastava et al., 2009; Khokon et al., 2010a,b). However, the mechanism how H_2_O_2_ is involved in NO production remains unknown. Cyclic AMP has been shown to be involved in LPS-induced NO production (Ma W. et al., 2009). Enzymatic mechanism mediated by nitrate reductases and NO synthase-like enzymes and non-enzymatic mechanism are involved in generation of NO in plant cells (Desikan et al., 2002; Moreau et al., 2008; Neill et al., 2008; Gayatri et al., 2013). Pharmacological studies have suggested that NO synthase-like enzymes were involved in flg22-, LPS-, and chitosan-induced stomatal closure (Melotto et al., 2006; Srivastava et al., 2009). However, the genes of NO synthase-like enzymes remain to be identified. It has been suggested that nitrate reductases were involved in chitosan-induced NO production (Srivastava et al., 2009). Two *Arabidopsis*, NIA1 and NIA2, were reported to be involved in ABA- and SA-induced NO production and stomatal closure (Desikan et al., 2002; Hao et al., 2010). Further investigation of the roles of NIA1 and NIA2 in MAMP-induced NO production and stomatal closure may move the field forward.

### Elevation of [Ca^2+^]_cyt_ Induced by MAMPs in Guard Cells

Cytosolic Ca^2+^ has long been considered as an important second messenger in guard cell signaling induced by various internal and external stimuli (Shimazaki et al., 2007; Kim et al., 2010; Roelfsema and Hedrich, 2010). A lot of methods have been introduced to monitor the [Ca^2+^]_cyt_ in guard cells including fluorescent dyes such as fura-2 and fluo-3, and genetically encoded indicators such as aequorin, yellow cameleon and R-GECO1 (Irving et al., 1992; Allen et al., 1999a,b; Harada and Shimazaki, 2009; Keinath et al., 2015). Among these methods, real-time imaging of live guard cells expressing the fluorescence resonance energy transfer-based indicator, yellow cameleon, has greatly advanced our understanding of Ca^2+^ signaling (Allen et al., 1999b; Mori et al., 2006). Based on this technique, we learn that spontaneous repetitive elevation of [Ca^2+^]_cyt_ occurs in guard cells, that stomata respond differently to different patterns of elevation of [Ca^2+^]_cyt_ (Allen et al., 2000, 2001), and that Ca^2+^ influx from the apoplast and Ca^2+^ releasing from internal stores are essential for the elevation of [Ca^2+^]_cyt_ induced by stimuli such as ABA and MeJA (Allen et al., 2000; Hamilton et al., 2000; Pei et al., 2000; Klüsener et al., 2002; Garcia-Mata et al., 2003; Munemasa et al., 2007; Akter et al., 2012; Hossain et al., 2014). Several MAMPs including chitosan, YEL, flg22 and chitin have been demonstrated to induce elevation of [Ca^2+^]_cyt_ in guard cells (Klüsener et al., 2002; Khokon et al., 2010a; Salam et al., 2013; Ye et al., 2013b; Thor and Peiter, 2014; Keinath et al., 2015). Pharmacological and genetic studies reveal that Ca^2+^ influx and Ca^2+^ release mechanisms are essential for elevation of [Ca^2+^]_cyt_ induced by chitosan, YEL and flg22 (Klüsener et al., 2002; Ye et al., 2013b; Thor and Peiter, 2014). Influx of Ca^2+^ is mediated by plasma membrane non-selective Ca^2+^-permeable cation channels (*I*_Ca_ channels), which are activated by hyperpolarization (Hamilton et al., 2000; Pei et al., 2000). It has been shown that LPS, chitosan and YEL activate *I*_Ca_ channels in guard cells (Klüsener et al., 2002; Ali et al., 2007; Ye et al., 2013b). The identification of *I*_Ca_ channels involved in MAMP signaling remains challenging. Studies have suggested that glutamate receptor-like channels (GLRs) are involved in elevation of [Ca^2+^]_cyt_ in seedlings induced by flg22, elf18, and chitin (Kwaaitaal et al., 2011) and that cyclic nucleotide-gated channels (CNGCs) are involved in elevation of [Ca^2+^]_cyt_ induced by LPS (Ali et al., 2007). On the other hand, recent studies suggest that neither GLRs nor CNGCs are essential for flg22-induced elevation of [Ca^2+^]_cyt_ (Thor and Peiter, 2014). Recently, OSCA1 and its homologs have been identified as an *I*_Ca_ channel involved in hyperosmolality response (Hou et al., 2014; Yuan et al., 2014). It remains unknown whether OSCA1 is involved in MAMP signaling. Pharmacological studies suggested that many factors involved in Ca^2+^ releasing in guard cells, such as NO, cGMP, cADPR, and IP3 (Klüsener et al., 2002; Garcia-Mata et al., 2003; Hossain et al., 2014). Recent studies showed that Ca^2+^ releasing is essential for flg22-induced elevation of [Ca^2+^]_cyt_ (Thor and Peiter, 2014). Application of NO does not activate *I*_Ca_ channels in the plasma membrane, but induces Ca^2+^ releasing, probably through cGMP-cADPR pathway (Garcia-Mata et al., 2003; Joudoi et al., 2013; Hossain et al., 2014). Therefore, it is likely that NO produced by MAMPs contributes to MAMP-induced elevation of [Ca^2+^]_cyt_ in guard cells. The identification of the transporters involved in Ca^2+^ releasing remains challenging.

### Sensing of Ca^2+^ in MAMP-induced Stomatal Movement

In *Arabidopsis*, there are a maximum of 250 proteins possibly having the Ca^2+^-binding EF-hand motif (Day et al., 2002), which play important roles in transducing the signal of Ca^2+^ to downstream events. In addition to the ubiquitous eukaryotic Ca^2+^ sensor calmodulin (CaM), other proteins containing EF-hand are CDPKs, CaM-like proteins (CML) and calcineurin B-like protein (CBL). CBLs can form complexes with CBL-interacting protein kinases (CIPKs) to convert the Ca^2+^ signal to phosphorylation events (Steinhorst and Kudla, 2013).

Ca^2+^-dependent protein kinases comprise a gene family of 34 members in *Arabidopsis*. The roles of the 34 CDPKs in plant growth and stress response can differ from each other due to their specificities in tissue expression, subcellular localization, [Ca^2+^]_cyt_ dependency, substrates and regulation mechanisms (Boudsocq and Sheen, 2013; Liese and Romeis, 2013). So far, several members of CDPKs including CPK3, CPK4, CPK5, CPK6, CPK8, CPK10, CPK11, and CPK23 are identified as positive regulators in stomatal movement induced by ABA, MeJA, CO_2_ and exogenous H_2_O_2_ (Mori et al., 2006; Zhu et al., 2007; Zou et al., 2010, 2015; Munemasa et al., 2011; Hubbard et al., 2012; Merilo et al., 2013; Brandt et al., 2015). Recent works showed that CPK6 positively functions in YEL-induced stomatal closure and inhibition of light-induced stomatal opening (Ye et al., 2013b). On the other hand, CPK6 shows a negative effect on H_2_O_2_ scavenging mechanism induced by YEL. Since the CPK6 also positively functions in stomatal closure induced by ABA and MeJA (Mori et al., 2006; Munemasa et al., 2011; Brandt et al., 2015), the results suggest that CPK6 is a convergent point of signaling for stomatal closure induced by abiotic and biotic stimuli. The important role of CPK6 is related to its regulation of S-type anion channels (see Activation of S-type Anion Channels in Response to MAMPs in Guard Cells). CDPKs including CPK6 are activated by flg22 and involved in flg22-induced H_2_O_2_ production in leaf disks and mesophyll cells (Boudsocq et al., 2010; Dubiella et al., 2013; Guzel et al., 2015). Recent studies showed that *cpk3 cpk6 cpk5 cpk11* loss-of-function mutation shows a trend to partly impair flg22-induced stomatal closure in a nanoinfusion experiment (Guzel et al., 2015). Nevertheless, the quadruple mutation did not strongly impair flg22-induced stomatal closure, which may attribute to the compensatory mechanism by other Ca^2+^ sensing mechanisms. A recent study showed that CPK28 negatively regulates flg22-induced stomatal closure and that BIK1 is a target of CPK28 (Monaghan et al., 2014). Results from *in vitro* experiments have shown that activation of CDPKs is dependent on [Ca^2+^]_cyt_ (Boudsocq et al., 2012). In ABA signaling, the activation of CDPKs including CPK6 seems to be solely dependent on the elevation of [Ca^2+^]_cyt_ induced by ABA (Laanemets et al., 2013; Brandt et al., 2015). On the other hand, the activation of CDPKs including CPK6 in response to flg22 is dependent on both elevation of [Ca^2+^]_cyt_ and FLS2-dependent signaling (Boudsocq et al., 2010; Dubiella et al., 2013). These results suggest the activation of CDPK differs in different signalings.

Research on involvement of CMLs and CBL-CIPK pairs in MAMP signaling in guard cells is scarce. In *Arabidopsis*, CML24 has been shown to be involved in LPS-induced stomatal closure, probably by regulating NO production (Ma et al., 2008; Walker, 2011). Recent studies have suggested that CBL-CIPK pairs are involved in stomatal response (Maierhofer et al., 2014). Future research is needed to identify CMLs and CBL-CIPKs involved in guard cell MAMP signaling.

Ca^2+^-sensing receptor (CAS) represents a Ca^2+^ sensor of low Ca^2+^ affinity/high-capacity that does not contain EF-hand motif and is associated with thylakoid membranes (Han et al., 2003; Nomura et al., 2008; Weinl et al., 2008). Studies have revealed that CAS is involved in stomatal closure induced by high extracellular Ca^2+^ and flg22, but not ABA (Han et al., 2003; Weinl et al., 2008; Nomura et al., 2012). In these studies, CAS has been shown to be important for elevation of [Ca^2+^]_cyt_ induced by high extracellular Ca^2+^ in guard cells and is required for flg22-induced elevation of [Ca^2+^]_cyt_ in *Arabidopsis* plants. These results suggest that Ca^2+^ releasing from chloroplast involving CAS is essential for flg22-induced stomatal closure.

### Open Stomata 1 Involvement in MAMP-induced Stomatal Closure

There are increasing results showing that OST1 plays a central role in guard cell signaling induced by various stimuli such as high CO_2_, low humidity, and ozone (Xie et al., 2006; Ache et al., 2010; Vahisalu et al., 2010; Xue et al., 2011; Merilo et al., 2013). Recent studies have revealed that OST1 kinase is also involved in stomatal closure induced by MAMPs including flg22, LPS, and YEL (Melotto et al., 2006; Montillet et al., 2013; Guzel et al., 2015; Ye et al., 2015). The importance of OST1 seems to be related to its essential role in activation of S-type anion channels (see Activation of S-type Anion Channels in Response to MAMPs in Guard Cells), because OST1 is not involved in elevation of [Ca^2+^]_cyt_ and H_2_O_2_ accumulation induced by YEL but is involved in S-type anion channel activation induced by ABA, CO_2_, YEL, and high Ca^2+^ (Xue et al., 2011; Hua et al., 2012; Brandt et al., 2015; Ye et al., 2015).

To our knowledge, there is no evidence that kinase activity of OST1 is increased by stimuli other than ABA in guard cells. On the other hand, recent studies have shown that flg22 does not increase OST1 kinase in *Arabidopsis* suspension cells and YEL does not increase OST1 kinase in *Arabidopsis* guard cells (Montillet et al., 2013; Ye et al., 2015). OST1 is mainly activated upon inhibition of PP2Cs by ABA perception in plants (Mustilli et al., 2002; Ma Y. et al., 2009; Park et al., 2009). Flg22 does not increase ABA content in plant and YEL does not induce transcription of ABA responsive gene, *RD29B*, in guard cells (Nomura et al., 2012; Ye et al., 2015), suggesting that flg22 and YEL do not increase ABA content in guard cells. These results taken together raise the possibility that resting activity of OST1 kinase, which itself is very weak, is involved in stomatal closure induced by stimuli including flg22 and YEL (**Figure 1**). Further research is needed to validate this possibility.

### Mitogen-activated Protein Kinase Involvement in MAMP-induced Stomatal Movement

Activation of MAPKs including MPK3, MPK4, and MPK6, is one of the early response induced by MAMPs such as flg22, elf18, LPS, and chitin (Zipfel et al., 2006; Boller and Felix, 2009; Ranf et al., 2015). It has been shown that MPK3 and MPK6 are essential for stomatal closure induced by flg22 and LPS (Gudesblat et al., 2009; Montillet et al., 2013). On the other hand, MPK3 and MPK6 seem not to be activated by ABA or involved in ABA-induced stomatal closure (Gudesblat et al., 2007; Montillet et al., 2013). Interestingly, MPK3 is involved in ABA inhibition of light-induced stomatal opening (Gudesblat et al., 2007), which is reminiscent of the involvement of OST1 in stomatal closure induced by flg22 and YEL. MPK4 is reported to be negatively involved in stomatal closure induced by *Pst* DC3000, in which flg22 is the dominant MAMP sensed by guard cells (Zeng and He, 2010; Hettenhausen et al., 2012). MPK9 and MPK12 function redundantly in stomatal closure induced by ABA, MeJA, YEL, and chitosan (Jammes et al., 2009; Salam et al., 2012, 2013). Further results reveal that mutation of *mpk9 mpk12* increased the susceptibility of *Arabidopsis* to spray-inoculated *Pst* DC3000 (Zeng and He, 2010; Jammes et al., 2011). On the other hand, it has been reported that MPK9 and MPK12 were not involved in stomatal closure induced by flg22 at 5 μM (Montillet et al., 2013). These results suggest that different MAMPs recruit different MAPKs to induce stomatal closure. MPK9 and MPK12 function redundantly in inhibition of light-induced stomatal opening induced by YEL while only mutation in MPK12 impaired ABA inhibition of light-induced stomatal opening (Salam et al., 2013; Des Marais et al., 2014). These results suggest that the regulation of MPK9 and MPK12 are differently regulated by ABA and YEL.

It has been reported that MPK3 and MPK6 are not involved in elevation of [Ca^2+^]_cyt_ induced by flg22 and elf18 and that MPK9 and MPK12 are not involved in elevation of [Ca^2+^]_cyt_ induced by YEL and chitosan (Salam et al., 2012, 2013). The activation of MAPKs can be induced by flg22 in a Ca^2+^-independent manner (Boudsocq et al., 2010; Ranf et al., 2011). These results suggest that MAPKs can function in parallel with Ca^2+^-dependent pathways to regulate stomatal movement induced by MAMPs. Note that activation of MAPKs can differs in mechanism among different MAMPs, as seen in recent studies that a RLCK, PBL27, is specifically required for activation of MAPKs by chitin but not flg22 (Shinya et al., 2014). MPK9 and MPK12 have been shown to be essential for activation of S-type anion channels induced by ABA, MeJA, and high extracellular Ca^2+^ (Jammes et al., 2009; Brandt et al., 2015; Khokon et al., 2015). It is likely that the function of MAPKs is related to regulation of S-type anion channels in MAMP-induced stomata movement.

### Regulation of Plasma Membrane Transporters in MAMP-induced Stomatal Movement

Guard cell volume is tightly regulated by transporters, especially the ones in plasma membrane. Recent studies have revealed that MAMPs regulate plasma membrane transporters, including *I*_Ca_ channels, *K*_in_ channels, S-type anion channels and H^+^-ATPases in guard cells (Klüsener et al., 2002; Zhang et al., 2008; Liu et al., 2009; Koers et al., 2011; Ye et al., 2013b, 2015; Guzel et al., 2015). In this section, we review these findings.

#### Activation of *I*_Ca_ Channels in Response to MAMPs in Guard Cells

*I*_Ca_ channels in the plasma membrane function as a pathway for Ca^2+^ influx, which is activated at hyperpolarization condition (Hamilton et al., 2000; Pei et al., 2000). Patch clamp results have shown that I_Ca_ channels are activated by ABA, MeJA, exogenous H_2_O_2_, and MAMPs including YEL, chitosan and LPS in *Arabidopsis* guard cells (Pei et al., 2000; Murata et al., 2001; Klüsener et al., 2002; Ali et al., 2007; Munemasa et al., 2011; Ye et al., 2013b, 2015). Intriguingly, activation of *I*_Ca_ channels is observed with cytosol dialyzed with ATP-free solution. These results suggest that MAMPs including YEL, chitosan and LPS can activate *I*_Ca_ channels in a phosphorylation-independent manner. Since application of H_2_O_2_ can activate *I*_Ca_ channels, it is likely that MAMP-induced H_2_O_2_ contributes to activation of *I*_Ca_ channels. YEL and chitosan activate *I*_Ca_ channels in guard cells that have most of the cell wall-bind peroxidases removed and LPS activate *I*_Ca_ channels in guard cells with cytosol dialyzed with NAD(P)H-free solution (Klüsener et al., 2002; Ali et al., 2007; Ye et al., 2013b). These results suggest that MAMPs can activate *I*_Ca_ channels in a H_2_O_2_-independent manner and/or that *I*_Ca_ channels activated by exogenous H_2_O_2_ are different from the ones by MAMPs in these experimental conditions (**Figure 1**). Further studies reveal that CPK6 is required for activation of *I*_Ca_ channels induced by ABA, MeJA, and YEL (Mori et al., 2006; Munemasa et al., 2011; Ye et al., 2013b). On the other hand, it has been shown that suppression of EF-hand-containing proteins by an inhibitor, W7, is required for LPS activation of *I*_Ca_ channels (Ali et al., 2007). These results suggest that Ca^2+^-dependent mechanism can play both negative and positive roles in activation of *I*_Ca_ channels induced by different MAMPs.

#### Activation of S-type Anion Channels in Response to MAMPs in Guard Cells

Early studies have identified two types of anion channels in guard cells, S-type and R-type anion channels (Schroeder and Hagiwara, 1989; Hedrich et al., 1990; Schroeder and Keller, 1992). While R-type anion channel is activated transiently and shows strong voltage dependency, S-type is weakly voltage-dependent and lack of time-dependent inactivation. Therefore, activation of S-type anion channel is likely to induce long-term anion eﬄux and sustained depolarization, representing a hallmark of stomatal closure. In *Arabidopsis* guard cells, several genes have been identified as S-type anion channels including SLAC1 and SLAC1 homolog 3 (SLAH3), and R-type channels, ALMT12. However, SLAC1 shows the most prominent role in regulation of stomatal closure induced by various stimuli, such as ABA, CO_2_, darkness and high extracellular Ca^2+^ (Negi et al., 2008; Vahisalu et al., 2008; Meyer et al., 2010; Sasaki et al., 2010). Electrophysiological studies have shown that S-type anion channels are activated by chitosan, YEL and flg22 in guard cells (Koers et al., 2011; Ye et al., 2013b, 2015; Guzel et al., 2015). Further results have shown that both SLAC1 and SLAH3 were required for flg22-induced stomatal closure and activation of S-type anion channel (Montillet et al., 2013; Guzel et al., 2015). It is unknown whether ALMT12 is involved in MAMP-induced stomatal movement. While the regulation R-type anion channels remains largely unknown, the regulation of S-type anion channels has been extensively studied. Here, we briefly review the regulation of SLAC1.

**FIGURE 2:**
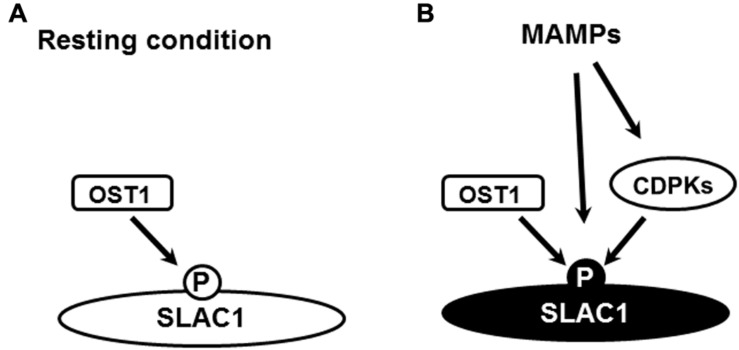
**Hypothetical regulation of SLAC1 by phosphorylation in guard cell MAMP signaling**. **(A)** Resting OST1 activity contributes to resting level of SLAC1 phosphorylation, particularly to the level of S120 phosphorylation, which is essential for the activation of SLAC1 by MAMPs. **(B)** In response to MAMPs, CDPK-dependent and –independent mechanisms change the phosphorylation status of SLAC1 including the phosphorylation of S59, resulting in SLAC1 activation. The change of color of letter “P” and “SLAC1” from black in **(A)** to white in **(B)** indicates a changed phosphorylation status and activation status, respectively.

In *Arabidopsis* guard cells, elevation of [Ca^2+^]_cyt_ is essential for S-type anion channel activation induced by ABA, high extracellular Ca^2+^ and CO_2_, but itself is not sufficient for activation of S-type anion channels (Allen et al., 2002; Siegel et al., 2009; Xue et al., 2011). These results raised the hypothesis that external and internal stimuli enhance/prime guard cells to respond to increased [Ca^2+^]_cyt_ levels and to activate S-type anion channels (Young et al., 2006; Siegel et al., 2009; Kim et al., 2010; Hubbard et al., 2012). Since the identification of SLAC1 as the main S-type anion channel in guard cells, many regulators have been revealed by *in vitro* experiments including CDPKs, CBL-CIPK complexes, OST1 and GUARD CELL HYDROGEN PEROXIDE-RESISTANT1 (GHR1) as positive regulators and PP2Cs as negative regulators (Geiger et al., 2009, 2010, 2011; Lee et al., 2009; Brandt et al., 2012, 2015; Hua et al., 2012; Scherzer et al., 2012; Maierhofer et al., 2014). However, only a few of the regulators have been shown to positively function in stomatal closure, including CPK3, CPK5, CPK6, CPK23, OST1, and GHR1 (Mustilli et al., 2002; Mori et al., 2006; Hua et al., 2012; Merilo et al., 2013; Brandt et al., 2015). In case of CPK23 there is also evidence that CPK23 negatively regulates salt and drought response (Ma and Wu, 2007). These results point out that the regulation of S-type anion channels can be complicated in guard cells in response to different stimuli. CPK6 and OST1 are required for stomatal closure induced by YEL and flg22 and activation by YEL of S-type anion channel (Melotto et al., 2006; Hua et al., 2012; Montillet et al., 2013; Ye et al., 2013b, 2015; Guzel et al., 2015). CPK6 does not seem to be engaged in activation of OST1 kinase by ABA and OST1 is not involved in [Ca^2+^]_cyt_ elevation in guard cells induced by YEL (Brandt et al., 2015; Ye et al., 2015). These results raise the possibility that CPK6 and OST1 directly regulate S-type anion channels in flg22 and YEL signaling as they do *in vitro*. Note that OST1 may function at resting activity. Further research on identification of phosphorylation sites of SLAC1 and their regulation by components such as CDPKs and OST1 is needed in order to further elucidate the regulation of S-type anion channels by MAMPs (**Figure 2**).

Studies have demonstrated that CPK6 and OST1 both can phosphorylate SLAC1 at Ser59 and Ser120 *in vitro*, which are probably dephosphorylated by PP2Cs (Geiger et al., 2009; Vahisalu et al., 2010; Brandt et al., 2012, 2015; Maierhofer et al., 2014). Recent studies showed that phosphorylation of Ser59 and S120 redundantly function in S-type anion channel activation by ABA in guard cells (Maierhofer et al., 2014; Brandt et al., 2015). On the other hand, mutation of Ser120 impaired stomatal closure induced by various stimuli other than ABA (Vahisalu et al., 2010; Merilo et al., 2013). These results suggest that different stimuli induce different phosphorylation pattern of S-type anion channels leading to stomatal closure. Further results showed that phosphorylation of Thr531 in SLAC1 results in constitutive activation of anion currents and phosphorylation of Ser59 and Ser120 are not sufficient for SLAC1 activation in oocytes (Maierhofer et al., 2014). These phosphorylation sites are strong candidates for the regulation by kinases and phosphatases in guard cells. The exist of different sites with different functions provides the molecular basis for Ca^2+^ priming model mentioned above.

#### Regulation of Potassium Channels and H^+^-ATPases by MAMPs in Guard Cells

Inward-rectifying K^+^ channels function as the main gate for K^+^ influx to the cytosol, while outward-rectifying K^+^ channels (*K*_out_ channels) function as the main gate for K^+^ eﬄux to the apoplast. Both *K*_in_ channels and *K*_out_ channels are voltage-dependent and do not inactivate with time (Schroeder, 1988; Blatt, 1990). The non-inactivation property of potassium channels allows long-term eﬄux of K^+^ needed during stomatal closure and influx of K^+^ needed during stomatal opening. *K*_out_ channels are activated but *K*_in_ channels are deactivated by depolarization of plasma membrane. In addition to the plasma membrane potential, flg22 suppressed both *K*_in_ channels, which was mediated by FLS2 and G-protein (Zhang et al., 2008). Recent studies also showed that YEL suppresses *K*_in_ channels, which is mediated by CPK6 (Ye et al., 2013b). Intriguingly, flg22 seems not to affect the voltage-dependency of *K*_in_ channels (Zhang et al., 2008), while ABA shifts the voltage dependency of *K*_in_ channels to more hyperpolarization (Armstrong et al., 1995). These results indicate that the suppression of *K*_in_ channel activity by ABA and MAMPs can be different in mechanism and suggest that decrease in number of active *K*_in_ channels contributes to the inhibition by MAMPs. It has been shown that KAT1, the dominant *K*_in_ channel in guard cells, undergoes internalization during stomatal closure (Sutter et al., 2007; Eisenach et al., 2012), and traffic system is important for flg22-induced stomatal closure (Spallek et al., 2013). Further studies suggested that phosphorylation of CDPK recognition sites in KAT1 by protein kinase C activator suppresses KAT1-mediated currents with voltage dependency unchanged (Sato et al., 2010). These results raise the possibility that phosphorylation by CPK6 of KAT1 contributes to YEL suppression of *K*_in_ channel activity. OST1 has been shown to phosphorylate KAT1 at Ter306 *in vitro*, which is critical for KAT1 activation in oocytes (Sato et al., 2009). It is likely that OST1 is involved in suppression of *K*_in_ channel activity by MAMPs. Flg22 also suppresses *K*_out_ channels in *Arabidopsis* guard cell (Zhang et al., 2008). It remains unknown whether this is common response for other MAMPs. Though activity of *K*_out_ channels is decreased, K^+^ eﬄux is sufficient for stomatal closure induced by flg22.

Plasma membrane H^+^-ATPases transport H^+^ into the apoplast at the expense of ATP, leading to hyperpolarization of plasma membrane, the driving force for stomatal opening in the light. It is known that ABA inhibits H^+^-ATPases in guard cells, which is essential for stomatal closure and inhibition of light-induced stomatal opening (Goh et al., 1996; Merlot et al., 2007). It has been shown that H_2_O_2_, NO, Ca^2+^, and phosphatidic acid contribute to ABA inhibition of H^+^-ATPases (Kinoshita et al., 1995; Zhang et al., 2004, 2007; Takemiya and Shimazaki, 2010; Uraji et al., 2012; Yin et al., 2013). Various MAMPs including flg22, chitosan, YEL and β-1→3-linked glucan inhibit light-induced stomatal opening. Studies have also shown that constitutive activation of AHA1 by *ost2* mutation, the dominant H^+^-ATPase in guard cells, impairs stomatal closure induced by flg22 and LPS (Liu et al., 2009). These results suggest that H^+^-ATPases are suppressed by MAMPs in guard cells.

### Involvement of Phytohormones in MAMP-induced Stomatal Response

It has been reported that stomatal closure induced by flg22 and LPS was impaired in ABA-deficient mutant (Melotto et al., 2006; Montillet et al., 2013; Du et al., 2014), suggesting endogenous ABA is involved in flg22 and LPS signaling in guard cells. This idea is supported by the fact that the master regulator of ABA signaling, OST1, is required for stomatal closure induced by flg22 and LPS. On the other hand, flg22 was shown not to induce ABA synthesis in *Arabidopsis* leaves and activation of OST1 in *Arabidopsis* suspension cells (Nomura et al., 2012; Montillet et al., 2013). These results suggest that resting level of ABA but not the elevating level of ABA induced by flg22 is required for signaling in guard cells. A possible function of the resting level of ABA is to produce the resting activity of OST1 (**Figure 1**). On the other hand, YEL and chitosan induced stomatal closure in ABA-deficient mutant (Issak et al., 2013). However, we may not be able to exclude the possibility that endogenous ABA is not required for stomatal closure induced by YEL and chitosan, since there is considerable ABA in these ABA-deficient mutants. For example the content of ABA in *aba2-2* is around 23% of those in wild type (Nambara et al., 1998). These results raise the possibility that the remained ABA is enough for stomatal closure induced by YEL and chitosan. Future work is needed to elucidate the role of endogenous ABA in stomatal closure induced by MAMPs.

Stomatal closure induced by LPS and *Pst* DC3000 is impaired in SA-deficient mutant (Melotto et al., 2006; Zeng and He, 2010), suggesting that endogenous SA is involved in stomatal closure induced by LPS and flg22. It seems that SA functions through NPR1, a master regulator of SA signaling, in flg22 signaling, since stomatal closure induced by *Pst* DC3000 and *Pst* DC3118 was impaired in *npr1-1* mutant (Zeng and He, 2010). Flg22 and LPS also induces SA production and expression of SA-responsive gene, *PR1*, in *Arabidopsis* leaves (Denoux et al., 2008; Tsuda et al., 2008; Nomura et al., 2012). A role of SA may be related to the regulation of PRR levels in guard cells, since recent works have shown that SA finely regulates levels of PRRs including FLS2 in *Arabidopsis* (Tateda et al., 2014). The endogenous SA may be recruited as a substrate for PRXs that are involved in flg22-indued stomatal closure (Mori et al., 2001). Future work is needed to elucidate how endogenous SA functions in guard cell MAMP signaling.

Studies have shown that ethylene signaling components, ETR1 and EIN2, are involved in flg22-induced stomatal closure probably by regulating FLS2 transcription and protein level and H_2_O_2_ production, but only in an unwounded condition (Mersmann et al., 2010). These results suggest that ethylene signaling is required for guard cell flg22 signaling in a stimulus-dependent manner. Ethylene production has been widely observed to be induced by various MAMPs including flg22, elf18, elf26, chitin, and YEL (Felix et al., 1991; Kunze et al., 2004; Zipfel et al., 2006). It is likely that ethylene signaling is also involved in stomatal closure induced by other MAMPs.

### Other Components Involved in MAMP Signaling in Guard Cells

Increasing evidences are emerging that reactive carbonyl species (RCS), such as 4-hydroxy-2-nonenal and acrolein, is produced by both enzymatic and non-enzymatic mechanisms and regulates guard cell signalings (Montillet et al., 2013; Islam et al., 2015). Recent studies have shown that RCS production mediated by a lipoxygenase (LOX), LOX1, is required for stomatal closure induced by flg22 but not ABA (Montillet et al., 2013). It remains unknown how the RCS is involved in flg22 signaling.

In addition to GHR1, recent studies identified several RLKs, including L-type lectin receptor kinase-VI.2 (LecRK-VI.2), LecRK-V.5, and Cysteine-rich receptor-like kinases (CRKs), which are involved in guard cell MAMP signaling. LecRK-V.5 has been reported to negatively regulate stomatal closure and H_2_O_2_ accumulation in guard cells induced by flg22, LPS, elf26 and ABA (Desclos-Theveniau et al., 2012). LecRK-VI.2 positively regulates stomatal closure induced by flg22 and elf26 but not ABA (Singh et al., 2012). Further results show LecRK-VI.2 is not involved in H_2_O_2_ production but activation of MAPKs induced by flg22 in leaves. Recent phenotypic studies of the T-DNA insertion mutants of CRK family have identified many of its members are involved in stomatal closure induced by stimuli including flg22, chitin and ABA, and shown that CRKs provide signaling specificity (Bourdais et al., 2015). For these RLKs, the future challenge is to elucidate their regulation and substrates in MAMP signaling.

## Redundancy in Guard Cell Mamp Signaling

Great advance in understanding guard cell signaling including the one induced by MAMPs has been made based on genetic methods, particularly using mutant plants. In these studies, functionally redundant mechanisms are suggested. Typical examples can be found in H_2_O_2_ production, sensing of Ca^2+^, MAPK function and regulation of anion channels. However, these mechanisms do have their own specificity. For example, CPK3 and CPK6 have different Ca^2+^ sensitivity, with CPK6 activated at lower Ca^2+^ concentration, but both can phosphorylate SLAC1 (Boudsocq et al., 2012; Scherzer et al., 2012; Laanemets et al., 2013). Theoretically, CPK6 functions at lower Ca^2+^ concentration, while CPK6 and CPK3 both function at higher concentration, which are therefore considered functionally redundant at higher concentration. It can be expected that functional redundancy appears depending on conditions and the strength of stimuli is an important factor to determine the occurrence of redundancy. A challenge for future dissection of signaling is to define the biological conditions and mimic them in the labs. It is also needed to mention that the functional redundancy in signaling should not be confused with compensatory mechanisms that have been widely observed in extreme experimental conditions for plants, such as constitutive loss-of-function mutations. It is likely that to change the properties of functionally redundant components is a common mechanism for compensation. For example, the gene expression level of *SLAH3* doubles in *slac1* mutant, which may account for the partial flg22-induced stomatal closure in *slac1* mutant (Geiger et al., 2011; Guzel et al., 2015). On the other hand, flg22-induced stomatal closure was abolished in *ost1* mutant in the same study but OST1 seems not to activate SLAH3. It is therefore possible that SLAC1 plays a dominant role but not function redundantly with SLAH3 in flg22-induced stomatal closure in wild-type plants. In the future, the challenge is to validate the contribution of these suggested redundant components to MAMP signaling in wild-type plants. Elucidation of the compensatory mechanisms in these mutants may also contribute to our understanding and is of particular importance in practical aspect.

## Comparison of Mamp Signalings in Leaf Epidermal Cells, Mesophyll Cells and Guard Cells

Unlike epidermal and mesophyll cells, guard cells do not have plasmodesmata but function autonomously. FLS2 and co-receptor, BAK1, are expressed in epidermal cells, mesophyll cells and guard cells, suggesting that the similar perception mechanism of flg22 exist in these three cell types (Robatzek et al., 2006; Shang et al., 2015). Future biochemical studies in a cell-specific context are needed to validate this suggestion. While many downstream components such as RbohD, CPK6, MPK3, and MPK6 seem to be expressed ubiquitously in the leaf, components, such as MPK9, MPK12, OST1, SLAC1, and ALMT12 are mainly expressed in guard cells (Mustilli et al., 2002; Mori et al., 2006; Negi et al., 2008; Vahisalu et al., 2008; Jammes et al., 2009; Meyer et al., 2010; Sasaki et al., 2010). Endogenous hormone ABA concentration is much higher in guard cells than epidermal and mesophyll cells (Waadt et al., 2014). These differences in signaling component levels determine the output of MAMP responses in different cell types. For example, flg22 induces H_2_O_2_ production and [Ca^2+^]_cyt_ elevation in guard cells, epidermal cells and mesophyll cells (Ranf et al., 2008; Jeworutzki et al., 2010; Desclos-Theveniau et al., 2012; Macho et al., 2012; Thor and Peiter, 2014; Guzel et al., 2015; Keinath et al., 2015). Flg22-induced depolarization of plasma membrane, the driven force of stomatal closure, is not impaired in *rboh*D, *slah3* and fusicoccin-treated mesophyll protoplasts, but flg22-induced stomatal closure is impaired in *rboh*D, *slah3*, and *ost2* plants, suggesting that flg22-induced depolarization of plasma membrane is different in mechanism in the two cell types (Jeworutzki et al., 2010; Macho et al., 2012; Kadota et al., 2014; Li et al., 2014; Guzel et al., 2015).

## Concluding Remarks and Outlooks

Since 2006, stomatal immunity has emerged as an important part of plant immunity. The output is stomatal closure and inhibition of stomatal opening to prevent microbe invasion. Though it is known that MAMPs are important signals to trigger stomatal immunity, we know little about how and how much MAMPs are exposed to the surveillance of guard cells. For example, stomatal closure by *Pst* DC3118 but not *E. coli* is abolished in *fls2* mutant (Melotto et al., 2006; Zeng and He, 2010), indicating that guard cells differently sense different pathogens even though it seems that they have the same set of MAMPs. It also remains unclear whether MAMPs including elf18/26, normally considered to exist inside the cells, are exposed to cell surface. A recent study suggests that *Arabidopsis* plants sense elongation factor Tu in the outer membrane vesicles secreted by Gram-negative bacteria (Bahar et al., 2016).

Though plasma membrane-localized PRRs in guard cells are widely accepted to function to perceive MAMPs, MAMP signaling also happens in the cell wall. It remains unknown whether the apoplast signaling is regulated by the plasm membrane-localized PRRs or there are PRRs in the apoplasts. Upon binding of PRRs and ligands, co-receptors are immediately recruited to form receptor complexes, which determine the specificity of signalings. The core signaling downstream to induce stomatal closure is that MAMP perception induces Ca^2+^-dependent mechanisms to activate S-type anion channels, which is dependent on Ca^2+^-independent mechanisms (**Figures 1** and **2**). Future research is needed to unravel new Ca^2+^-dependent mechanisms. Emergent challenge is to elucidate how Ca^2+^-independent mechanisms, such as the ones mediated by OST1 and MAPKs, are involved in MAMP signaling in guard cells. Signaling specificity provided by RLCKs and CRKs has been observed in MAMP-induced stomatal response. Further elucidation of these specificities is essential for dissecting MAMP signaling in guard cells.

The roles of phytohormones in guard cell MAMP signaling are still unclear. Though increase of phytohormone by methods such as direct application can induce stomatal movement, it is less likely that MAMPs increase the level of phytohormone to trigger stomatal movement, as seen in the case that flg22 does not increase ABA content. Therefore, it is important to note that the function of elevating level of phytohormone can be different from that of phytohormone in unstressed plants. An emerging role is that phytohormones, such as ethylene and SA, in unstressed plants regulate the level of PRRs. A possible role is that phytohormones, such as ABA, are important for providing basal activity of important signaling components. The future challenge is to elucidate the roles of endogenous phytohormones in guard cell MAMP signaling.

## Author Contributions

All authors listed, have made substantial, direct and intellectual contribution to the work, and approved it for publication.

## Conflict of Interest Statement

The authors declare that the research was conducted in the absence of any commercial or financial relationships that could be construed as a potential conflict of interest.
